# Appropriate Delivery of the CRISPR/Cas9 System through the Nonlysosomal Route: Application for Therapeutic Gene Editing

**DOI:** 10.1002/advs.201903381

**Published:** 2020-06-13

**Authors:** Hang Yin, Xiaoling Yuan, Lihua Luo, Yichao Lu, Bing Qin, Junlei Zhang, Yingying Shi, Chunqi Zhu, Jie Yang, Xiang Li, Mengshi Jiang, Zhenyu Luo, Xinyu Shan, Dawei Chen, Jian You

**Affiliations:** ^1^ College of Pharmaceutical Sciences Zhejiang University Hangzhou 310058 P. R. China; ^2^ School of Pharmacy Shenyang Pharmaceutical University Shenyang 110016 P. R. China

**Keywords:** CDC6, CRISPR/Cas9, endoplasmic reticulum, gene editing, pardaxin

## Abstract

The development of gene delivery has attracted increasing attention, especially when the introduction and application of the CRISPR/Cas9 gene editing system appears promising for gene therapy. However, ensuring biosafety and high gene editing efficiency at the same time poses a great challenge for its in vivo applications. Herein, a pardaxin peptide (PAR)‐modified cationic liposome (PAR‐Lipo) is developed. The results are indicative that significantly enhanced gene editing efficiency can be obtained through the mediation of PAR‐Lipos compared to non‐Lipos (non‐PAR‐modified liposomes) and Lipofectamine 2000, owing to its protection toward carried nucleotide by the prevention of lysosomal capture, prolongation of retention time in cells through the accumulation in the endoplasmic reticulum (ER), and more importantly, facilitation of the nuclear access via an ER‐nucleus route. Accumulation of PAR‐Lipos in the ER may improve the binding of Cas9 and sgRNA, thus further contributing to the eventually enhanced gene editing efficiency. Given their high biosafety, PAR‐Lipos are used to mediate the knockout of the oncogene CDC6 in vivo, which results in significant tumor growth inhibition. This work may provide a useful reference for enhancing the delivery of gene editing systems, thus improving the potential for their future clinical applications.

## Graphical abstract

Pardaxin modification enables the cationic liposomes to easily escape capture by lysosomes, enter the nucleus via an ER (endoplasmic reticulum)–nucleus route, and protects the DNA cargo from damage to enable further delivery of the cargo to the ER, inducing increased DNA entry into the nucleus to enhanced gene editing.

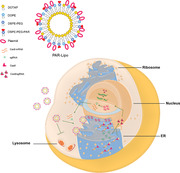

The revolutionary gene editing system named CRISPR (clustered regularly interspaced short palindromic repeats) is derived from the adaptable immune mechanisms of bacteria and archaea.^[^ ^1^ ^]^ The CRISPR/Cas system is a powerful combination of RNA‐guided DNA endonuclease Cas9 and a chimeric single guide RNA (sgRNA).^[^ ^2^ ^]^ The Cas9‐sgRNA combination can induce precise double‐strand DNA breaks (DSBs) by recognizing the protospacer‐adjacent motif adjacent to a target gene.^[^ ^3^ ^]^ Then, the DSBs trigger targeted disruption and result in loss‐of‐function mutations at specific genomic loci.^[^ ^4^ ^]^ A widely used delivery form of the CRISPR/Cas system is a plasmid encoding Cas9 nuclease and a targeted sgRNA sequence (CRISPR/Cas9), which exhibits great potential in gene‐associated disease therapies such as cancer therapy.^[^ ^5^ ^]^

However, the expanding applications of the CRISPR/Cas9 system remain restricted by its large size, which typically results in low DNA editing efficiency.^[^ ^6^ ^]^ In addition to the large size of the system, the biosafety of its delivery in vivo is also a considerable concern. Common viral carriers, including adeno‐associated viral, adenoviral (Ad), and lentiviral vectors, have been used to deliver the CRISPR/Cas9 system.^[^ ^7^ ^]^ The limited loading capacity of the CRISPR/Cas9 system and the high immunogenicity of these carriers may greatly limit the further application of the gene editing strategy. As an alternative, non‐viral delivery systems may exhibit greater advantages for application in vivo due to the higher biosafety and more controllable preparation of these systems than viral vector systems.^[^ ^8^ ^]^

Over recent decades, the application of non‐viral gene delivery systems has attracted increasing attention, especially biomaterial‐based functional vector systems.^[^ ^9^ ^]^ However, the use of these non‐viral carriers still faces several barriers, such as insufficient cellular uptake, difficult endosomal escape, and poor nuclear gene delivery capacity, resulting in a low transcription efficiency.^[^ ^10^ ^]^ When non‐viral vectors are used to deliver the CRISPR/Cas9 system, the barriers also limit the efficiency of gene editing, which depends on the transcription of Cas9 and the nuclear access of Cas9‐sgRNA. The endoplasmic reticulum (ER), a continuous membrane system, includes the nuclear envelope (NE) and the peripheral ER. The NE, the major barrier of the nucleus, consists of two lipid bilayers, the inner nuclear membrane and the outer nuclear membrane, and shares a common lumen with the peripheral ER.^[^ ^11^ ^]^ The rough sheets of the peripheral ER, defined by the high density of ribosomes on their cytosolic surfaces, are the main sites of protein synthesis.^[^ ^12^ ^]^ The intimate relationship between the ER and the nuclear membrane may enable an ER–nucleus pathway for component transport.^[^ ^13^ ^]^ We hypothesize that the CRISPR/Cas9 system may enable higher DNA editing efficiency by skipping lysosomal capture and entering the nucleus via an ER–nucleus route.

Herein, we developed a novel cationic liposome containing 1,2‐dioleoyl‐3‐trimethylammoniumpropane (DOTAP), a cationic lipid, for delivery of the CRISPR/Cas9 gene editing system. The liposome was further surface‐modified with a cationic peptide (pardaxin [PAR], **Figure** **1**A) to circumvent the lysosomal pathway and localize the liposome to the ER after cellular internalization. A plasmid DNA encoding the CRISPR/Cas9 system was employed to load the PAR‐modified liposomes (PAR‐Lipo) and test their gene editing efficiency. Furthermore, antitumor effects were investigated by assessing cleavage of the oncogene hCDC6, a licensing factor for the strict control of DNA replication, upon liposomal delivery of the CRISPR/Cas9 system. Our study provides a valuable reference for in vivo gene editing via non‐viral vectors and its application in tumor therapy.

**Figure 1:**
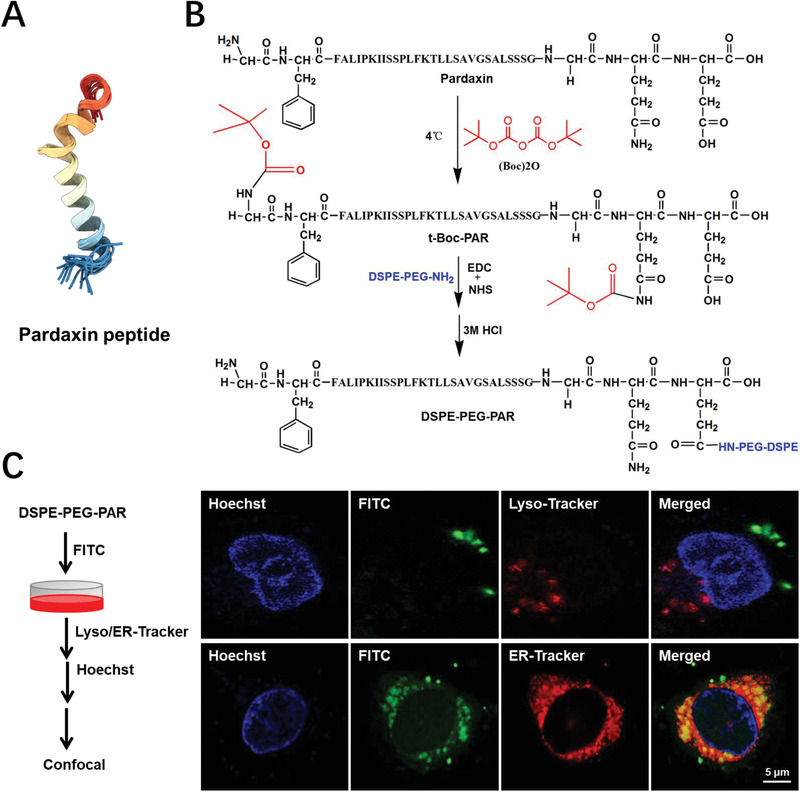
The synthesis and intracellular localization of DSPE‐PEG‐PAR after 2 h incubation with MCF‐7 cells. A) The spatial structure of the pardaxin peptide. The structure image was obtained from the RCSB Protein Data Bank (https://www.rcsb.org/). B) The organic synthetic routes of DSPE‐PEG‐PAR. C) The intracellular co‐localization images of DSPE‐PEG‐PAR with lysosomes and ER were obtained by confocal microscopy, bar: 5 µm.

The conjugate of DSPE‐PEG‐PAR was first synthesized by a condensation reaction of the amino groups in DSPE‐PEG_2000_‐NH_2_ with the carboxyl groups in PAR (Figure 1B), and synthesis was confirmed by ^1^H‐NMR and IR spectroscopy (Figure S1, Supporting Information). To investigate the performance of the conjugate in lysosome escape, fluorescein isothiocyanate (FITC) was chosen to label DSPE‐PEG‐PAR (green). LysoTracker (red) and Hoechst (blue) staining showed the intracellular positions of lysosomes and nuclei, respectively (Figure 1C). The images show that the conjugate did not localize to the same positions as the lysosomes after 4 h of incubation, suggesting that this conjugate created via PAR modification can escape capture by lysosomes and thus represents a nonlysosomal intracellular route. Furthermore, high colocalization was observed between the signal of the conjugate and the ER (Figure 1C), which demonstrated ER‐targeted accumulation of DSPE‐PEG‐PAR after cellular internalization. It was expected that liposomes with the DSPE‐PEG‐PAR modification might also be able to successfully evade the lysosomal pathway for the protection of the DNA cargo and accumulate in the ER.

PAR‐Lipos with different PAR modifications from the addition of DSPE‐PEG‐PAR and different lipid ratios (Table S1, Supporting Information) were prepared through thin‐film hydration. Table S2, Supporting Information, shows the sizes and polymer dispersity index of PAR‐Lipos and their complexes with plasmids (mass ratio of 5:1). Upon comparing the particle size changes and stability before and after the formation of the complexes, the optimal formulation range was determined to be limited to liposome No. 2–4 (Table S2, Supporting Information). The uptake of these complexes with DiD labeling after 4 h of incubation with MCF‐7 cells was observed, and liposome 3 presented the highest internalization level (DOTAP:DOPE ratio of 4:1 and DSPE‐PEG:DSPE‐PEG‐PAR ratio of 1:3) (Figure S2, Supporting Information); liposome 3 which then chosen as the optimized formulation of PAR‐Lipo for the next study.

The morphologies of PAR‐Lipos and non‐Lipos were observed by transmission electron microscopy (TEM) and showed complete membranes and spherical structures with diameters of 50–100 nm (**Figure** **2**A). Although the average particle sizes of the complexes formed by PAR‐Lipos and pSpCas9‐sgRNA were slightly larger at approximately 80 nm than those of PAR‐Lipos alone at approximately 70 nm, as determined by dynamic light scattering, their size distributions were narrower (Figure 2B). Similar results were obtained for non‐Lipos as controls without PAR modification.

**Figure 2:**
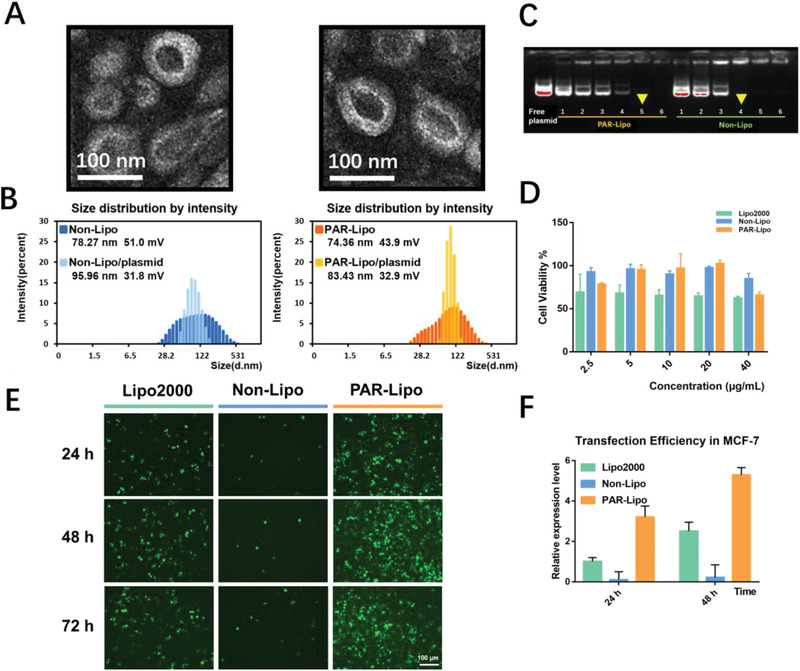
Characterization and transfection in vitro of the complexes formed by liposomes and plasmid. A) TEM images of non‐Lipo and PAR‐Lipo. Scale bars, 100 nm. B) Particle size and zeta potential of non‐Lipo and PAR‐Lipo and their complexes with plasmid (liposome/plasmid of 5:1, weight ratio). C) Agarose gel electrophoresis assay of complexes formed by non‐Lipo and PAR‐Lipo with plasmid (liposome/plasmid = *n*:1, *n* = 1–6, weight ratio). D) Cytotoxicity against MCF‐7 cells was evaluated by MTT after the incubation of various liposomes for 48 h (*n* = 6). E) EGFP transfection in MCF‐7 cells after 24, 48, and 72 h incubation with the complexes of Lipo2000/pEGFP (1.5:1), non‐Lipo/pEGFP (5:1), and PAR‐Lipo/pEGFP with (5:1). F) The relative expression level of EGFP fluorescence was obtained by the analysis using ImageJ software. All error bars represented the ± SD.

An agarose gel electrophoresis assay was performed to assess the cargo capacity of the vectors, showing that complete, tight complexes could be formed from PAR‐Lipos and pSpCas9‐sgRNA at weight ratios of over 5:1 (Figure 2C). It was found that non‐Lipos could form tight complexes with the plasmid only at a ratio of 4:1, suggesting that PAR modification on the liposome surface may induce some steric hindrance for binding between liposomes and the plasmid.

The cytotoxicity of the various liposomes was assessed in MCF‐7 and Huh7 cells by MTT assay. There was no significant difference in cytotoxicity between PAR‐Lipos and non‐Lipos in the concentration range of 2.5–20 µg mL^−1^ against MCF‐7 cells (Figure 2D) and in the concentration range of 2.5–40 µg mL^−1^ against Huh7 cells (Figure S3, Supporting Information) after 24 and 48 h of incubation. However, Lipo2000 clearly caused significantly more severe cell growth inhibition than PAR‐Lipos in the middle concentration range of 5–20 µg mL^−1^ in MCF‐7 cells and in the high concentration range of 20–40 µg mL^−1^ in Huh7 cells. The results suggest that PAR‐Lipos may have increased biosafety compared to that of Lipo2000, a conventional commercial transfection reagent.

Gene transfection activity greatly determines the efficiency of gene editing. Thus, a PAR‐Lipo‐mediated in vitro transfection experiment was performed using EGFP as a reporter gene. Interestingly, compared with non‐Lipos, PAR‐Lipos resulted in much higher GFP fluorescence in MCF‐7 cells, with a 20‐fold increase after 24, 48, or 72 h of incubation (Figure 2E,F). PAR‐Lipos even exhibited significantly stronger transfection activity than Lipo2000. The highest transfection efficiency in Huh‐7 cells was also observed to be mediated by PAR‐Lipos (Figure S4A,B, Supporting Information). Clearly, the PAR surface modification leads to a significant increase in the gene transfection activity of cationic liposomes, which may be related to the unique intracellular transport pathway of the liposomes caused by PAR after cell internalization.

HEK293T cells with stable GFP expression (HEK293T‐GFP) and a plasmid encoding the CRISPR/Cas9 system with a KO target of GFP (pSpCas9‐sgGFP, Figure S4C, Supporting Information) were first employed here. For gene editing, the cells were treated with complexes of PAR‐Lipos or Lipo2000 and plasmid according to the procedures in **Figure** **3**A. The GFP fluorescence was significantly reduced in cells treated with the PAR‐Lipo complexes compared to cells treated with Lipo2000 complexes, and the degree of reduction increased with treatment time from 24 to 72 h (Figure 3B). Clearly, PAR‐Lipos caused more significant GFP fluorescence disappearance than Lipo2000 when the PAR‐Lipo/plasmid ratio was 5–11:1. Flow cytometry analysis indicated that the efficiency of GFP KO for the PAR‐Lipo‐formed complexes (5:1) was over 60% and over threefold higher than that of the complexes formed by Lipo2000 after 72 h of transfection (Figure 3C,D). Interestingly, increasing the PAR‐Lipo/plasmid mass ratio could further enhance the KO effect, yielding KO efficiencies ranging from approximately 10% to over 80% for complex ratios from 5:1 to 11:1 after 24 h of transfection. However, considering the possible potential toxicity of high doses of PAR‐Lipos, a mass ratio of 7:1 for the complexes was chosen as the final optimized option. The gene editing of GFP was further demonstrated by DNA agarose gel electrophoresis. A clear band for the GFP fragment was obtained (Figure 3E), indicating successful cleavage of GFP DNA. Clearly, compared to Lipo2000, PAR‐Lipos induced greater GFP‐editing effects (Figure 3F).

**Figure 3:**
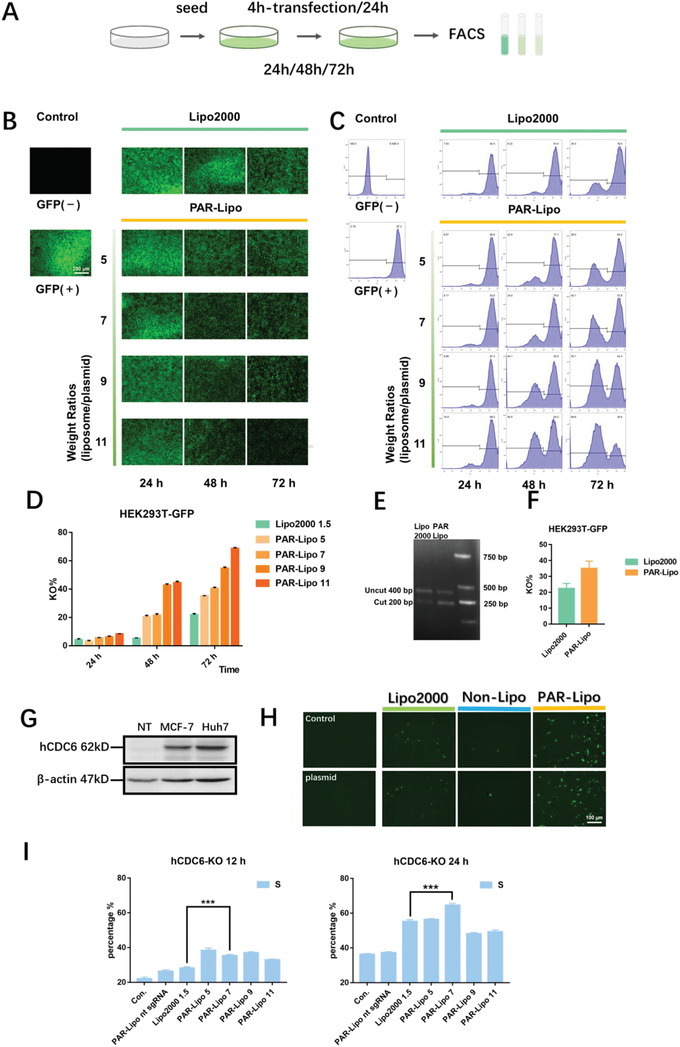
In vitro gene editing efficiency mediated by PAR‐Lipo formed complexes. A) The procedure of in vitro gene editing using CRISPR/Cas9 system. HEK293T‐GFP cells were transfected by complexes for 24, 48, and 72 h, followed by flow cytometry assessment. B) GFP expression was observed by fluorescence microscope after in vitro gene editing mediated by Lipo2000/plasmid complexes (1.5:1) and PAR‐Lipo/plasmid complexes (*n*:1, *n* = 5–11). C) Flow cytometry analysis of GFP gene editing in HEK293T‐GFP cells transfected by the complexes. D) Quantitative analysis of flow cytometry. E) Agarose gel electrophoresis results via T7E1 assay after 72 h transfection by Lipo2000/plasmid complexes (1.5:1) and PAR‐Lipo/plasmid complexes (5:1). F) Quantitative assay of agarose gel electrophoresis results using ImageJ software. G) The expression level of CDC6 in MCF‐7 and Huh7 cells (NT: normal tissue). H) GFP expression level in MCF‐7 cells after 72 h transfection by plasmid, Lipo2000/pSpCas9‐sgCDC6 complexes (1.5:1), and PAR‐Lipo/pSpCas9‐sgCDC6 complexes (5:1), MCF‐7 cells without any treatment was used as a control (Con.). I) The percentage of MCF‐7 cells in S phase, as measured by flow cytometry after 12 and 24 h gene editing to CDC6 (Con.: control without any treatment, PAR‐Lipo nt sgRNA: control CRISPR/Cas9 plasmid containing the non‐target sequence). All error bars represented the ± SD.

Furthermore, a CRISPR/Cas9 system encoding the GFP reporter gene with a KO target of CDC6 (pSpCas9‐sgCDC6, Figure S4C, Supporting Information) was used as a therapeutic plasmid to test the cancer cell‐killing effect of gene editing. Huh7 and MCF‐7 cells exhibited high expression of CDC6 (Figure 3G), which is crucial for their high rates of proliferation. Figure 3H shows that there was significantly higher GFP expression in Huh7 cells after 24 and 48 h of transfection with PAR‐Lipo‐formed complexes than after transfection with Lipo2000 or non‐Lipo‐formed complexes, which suggested successful gene editing of CDC6. The cells with editing presented significantly extended S phases compared to the cells without editing (Figure 3I) as a response to the stagnation of DNA replication due to CDC6 KO.

PAR‐Lipos and non‐Lipos with DiD fluorescent labeling as well as their complexes with a representative plasmid (pSpCas9‐sgGFP) presented obvious cellular internalization after 4 h of incubation, with no significant difference between the vectors with and without PAR modification (**Figure** **4**A). The results suggested that the enhanced gene editing effect mediated by PAR‐Lipos is attributable to its unique intracellular transport behavior.

**Figure 4:**
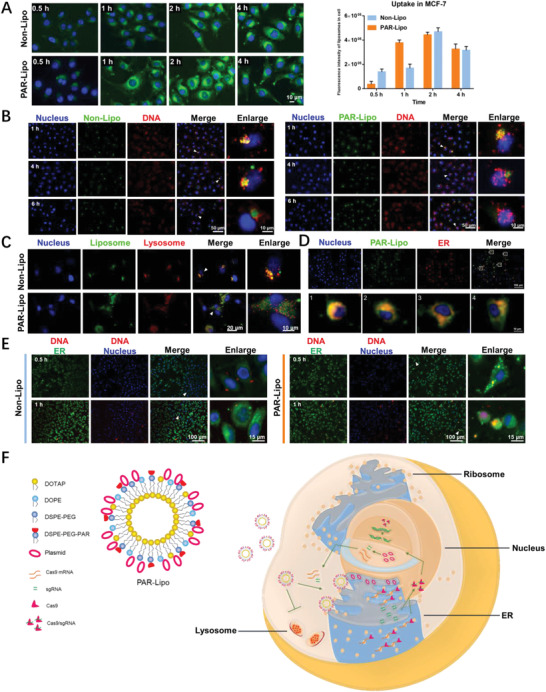
The intracellular transport behavior of PAR‐Lipo. A) The cell internalization level of DiD labeled PAR‐Lipo and non‐Lipo. B) The intracellular behavior of DNA cargos mediated by PAR‐Lipo and non‐Lipo. C) The subcellular localization of liposomes with lysosome. D)The co‐localization of PAR‐Lipo with ER. E) The intracellular distribution of free Cy3‐DNA in cells after a pre‐incubation with the DNA, followed by the treatment with PAR‐Lipo or Non‐Lipo. F) The possible mechanism for enhanced gene editing efficiency mediated by PAR‐Lipo. PAR‐Lipo mediated CRISPR/Cas9 system to skip the lysosomes capture and deliver it into ER, inducing an increased entrance of the system into nucleus via an ER‐nucleus route. All error bars represented the ± SD.

To monitor the intracellular behavior of the plasmid mediated by the liposomes, Cy3‐labeled DNA was mixed with the representative plasmid and then used to form complexes with the liposomes. It was first found that the complexes formed by PAR‐Lipos could cause DNA to reside in cells for a longer time than the complexes formed by non‐Lipos after internalization and that the PAR‐Lipo complexes induced significantly more DNA entry into the nuclei after 6 h of transfection (Figure 4B). The ER and lysosomes were further labeled with ER‐Tracker and LysoTracker, respectively. Most of the complexes formed by non‐Lipos entered lysosomes after their internalization. However, no colocalization was observed between the fluorescence of the complexes formed by PAR‐Lipos and lysosomes, indicating that the complexes did not follow the conventional lysosomal‐endosomal pathway (Figure 4C). The high colocalization of fluorescence with the ER demonstrated that PAR‐Lipos could selectively deliver DNA into the ER around the nucleus (Figure 4D), which may have contributed to the increased DNA entry into nuclei.

The entry of the plasmid encoding the CRISPR/Cas9 system into the nucleus is critical for the expression of sgRNA and the nuclease Cas9. Successful gene editing also relies on the binding of sgRNA to Cas9, which then re‐enters the nucleus to target the cleavage site. In traditional nonviral vector‐mediated gene transfection, an excessively high ratio of vector to DNA plasmid easily results in a decrease in transfection efficiency, mainly because excessive positive charge hinders the release of the plasmid from the formed complexes. We found that further increasing the ratio of PAR‐Lipos to pSpCas9‐sgGFP resulted in a significantly higher gene editing efficiency for GFP (Figure 3B–D) that may have be related to the accumulation of PAR‐Lipos in the ER, thereby promoting the binding of sgRNA to Cas9. A Cy3‐labeled small DNA fragment that was internalized into the cells was used to simulate sgRNA successfully expressed by the CRISPR/Cas9 system, and the cells were further incubated with PAR‐Lipos to study the effect of the vector on the intracellular behavior of sgRNA. Figure 4E indicates that the fluorescence of Cy3 was first concentrated in the ERs of the cells treated with PAR‐Lipos, while the fluorescence was widely distributed throughout the cells treated with non‐Lipos. A possible explanation for this finding is that large numbers of positively charged PAR‐Lipos in the ER cause the accumulation of DNA at the ER. These results suggest that sgRNA expressed by the CRISPR/Cas9 system may also be able to accumulate at the ER, where it may be easier for the sgRNA to bind with Cas9 theoretically translated in the ER and return to the nucleus (as described in Figure 4F), resulting in enhanced gene editing efficiency.

Statistical analysis of clinical data indicated that the expression levels of hCDC6 (human CDC6) in breast cancer (BRCA) and liver cancer (LIHC) were significantly higher than those in corresponding normal tissues (**Figure** **5**A). The survival curves of patients with breast and liver cancer are shown in Figure 5B and indicate a longer survival period for patients with lower hCDC6 expression. Considering the key role of the hCDC6 gene in the replication and proliferation of various cancer cells, the potential therapeutic effect of hCDC6 knockout via mediation with PAR‐Lipos was evaluated in vivo. Mice bearing MCF‐7 tumors were intravenously injected with saline, naked plasmid (pSpCas9‐sgCDC6), Lipo2000/plasmid complexes (at a mass ratio of 1.5:1), or PAR‐Lipo/plasmid complexes (at a mass ratio of 7:1) according to the protocol in Figure 5C. The mice injected with the PAR‐Lipo/plasmid complexes clearly exhibited the strongest tumor growth inhibition (Figure 5D) and the smallest average tumor weights after the treatments (Figure 5E). Compared with Lipo2000/plasmid complexes or naked plasmids, the PAR‐Lipo/plasmid complexes caused greater downregulation of cancer cell proliferation and resulted in fewer metastatic cancer cells in the liver, as determined by a Ki67 staining assay (Figure S5A, Supporting Information). The results were likely attributable to the higher CDC6 KO efficiency of the PAR‐Lipo complexes than of the other treatments in cancer cells, which was further confirmed by a western blot assay that indicated the lowest expression of CDC6 in the PAR‐Lipo complex‐treated group (Figure 5F). Importantly, CDC6 KO did not cause significant discomfort to the animals compared to saline treatment. The animals in all groups demonstrated high rates of survival throughout the experiment, and there were no significant differences in the histological morphology of vital organs (Figure S5B, Supporting Information) or in the body weight change trends among groups (Figure S5C, Supporting Information).

**Figure 5:**
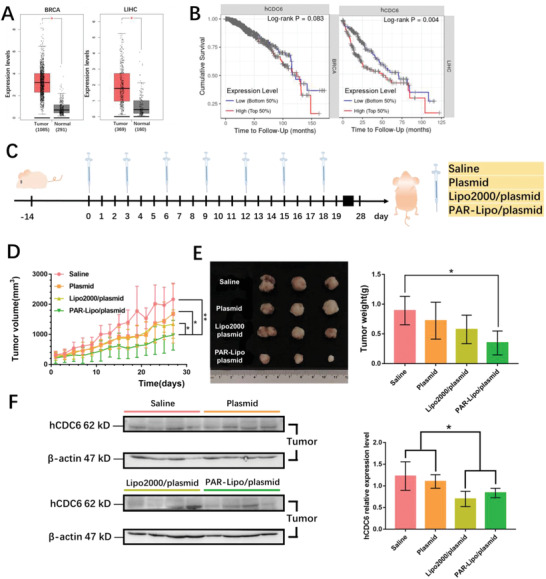
In vivo antitumor effect by CDC6 knockout. A) Gene expression of hCDC6 between tumor and normal tissues of patients, which was obtained from the GEPIA (http://gepia.cancer‐pku.cn/). B) Survival curves of patients with different expression of hCDC6 gene, which were available from the TIMER (https://cistrome.shinyapps.io/timer/). C) The scheme of in vivo gene editing to CDC6 mediated by PAR‐Lipo. Orthotopic breast cancer model was established by injecting MCF‐7 cells into the breast pad of nude mice. After 14 days, the mice were treated with saline, naked plasmid, Lipo2000/plasmid complexes, and PAR‐Lipo/plasmid complexes, respectively, according to the schedule. D) The curves show the changes of tumor volume during the treatments, *n* = 6. E) The representative tumor imaging and their mean weight isolated from mice at the end of experiment. *p < 0.05, **p < 0.01. F) CDC6 expression level of representative tumors was tested through western blot assay after the treatments. All error bars represented the ± SD.

The biodistribution of the PAR‐Lipo/plasmid complexes was investigated after intravenous injection, and the results indicated that the complexes were mainly distributed in the liver, with only minor distribution in the tumors (Figure S6, Supporting Information). Therefore, an orthotopic liver cancer model was established by injecting Huh7‐Luc tumor blocks into mouse livers. The therapeutic effect of CDC6 KO mediated by PAR‐Lipos against liver cancer was further evaluated according to the same protocol (Figure 5C). Luciferase bioluminescence imaging of the mice in each group was performed at day 0 and day 21 after the first injection (**Figure** **6**A) and indicated a significant reduction in mean fluorescence intensity with prolonged treatment time for the group treated with PAR‐Lipo/plasmid complexes. However, the average fluorescence became stronger to varying degrees in the mice in the saline, naked plasmid, and Lipo2000/plasmid groups (Figure 6B). The results indicated that PAR‐Lipo‐mediated gene editing induced more effective CDC6 KO in cancer cells than other types of gene editing, resulting in stronger growth inhibition of the orthotopic liver tumors. The average body weights of the animals and the histological morphology of vital organs did not significantly change in any of the groups (Figure 6C; Figure S7, Supporting Information), suggesting the good biosafety of the CDC6 KO treatments.

**Figure 6:**
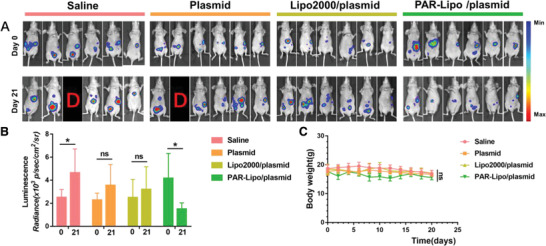
In vivo gene editing to CDC6 mediated by PAR‐Lipo against Huh7‐Luc tumors (*n* = 6). A) Luciferase bioluminescence images of Huh7‐Luc tumor before and after the various treatments (D means dead). B) The mean bioluminescence intensity in each group. C) The body weight curves of mice in each group. All error bars represented the ± SD.

According to the central principle of genetic information transmission from DNA to RNA to proteins, DNA, as the carrier of genetic information, determines all the physiological processes in an organism. Diseases often manifest as abnormalities at the protein level, while gene therapy occurs upstream of proteins, namely, at the levels of DNA and RNA, to ameliorate abnormalities in phenotype by changing the genotype.^[^ ^14^ ^]^ Gene editing, the most accurate and efficient means to modify genes, is considered a fundamental strategy to treat diseases, especially those caused by congenital genetic defects.^[^ ^15^ ^]^ In recent years, precise gene editing in cells in vitro through physical introduction or viral vectors has been performed. However, these gene editing strategies are difficult to apply in vivo due to the damage they cause to normal cells or due to their potential immunogenicity.^[^ ^16^ ^]^

Due to their higher biosafety, nonviral vectors have greater potential for gene delivery in vivo than viral vectors but tend to exhibit low transfection efficiency, mainly because they are easily captured by lysosomes after cellular internalization.^[^ ^17^ ^]^ We found that functional modification of nonviral vectors may be an effective method to improve their transfection efficiency. Herein, PAR peptide modification was selected as a strategy for assigning nonlysosomal transport pathways to cationic liposomes. Our data indicated that PAR‐Lipos could help the gene editing system bypass lysosomes (Figure 4C), enabling escape of the system from lysosomal capture and thus protecting the DNA cargo from damage. In addition, PAR‐Lipos could deliver the system to the ER (Figure 4D) and effectively prolong the retention time of DNA in cells (Figure 4B). We believe that ER localization of the CRISPR/Cas9 plasmid editing system may improve Cas9 expression via an ER‐nucleus route. A similar study reported that gene transfection efficiency can be enhanced by increasing plasmid entry into nuclei via an ER route.^[^ ^18^ ^]^ Importantly, PAR‐Lipos may promote sgRNA aggregation at the ER (Figure 4E) and subsequent binding to Cas9, which is translated in ribosomes of the ER, resulting in efficient entry of the Cas9‐sgRNA complex into the nucleus for gene editing. As a result, PAR‐Lipos mediated significantly greater gene editing efficiency in vitro than non‐Lipos or Lipo2000 (Figure 3) and exhibited a markedly stronger antitumor effect when used for delivery of the gene editing system for knockout of an oncogene (Figures 5, 6).

Pardaxin is a membrane‐penetrating peptide originally isolated from the fish *Pardachirus marmoratus*. The most studies of polypeptide‐modified vectors focused on increasing their accumulation in special tissues (e.g., tumors), but few studies involved in targeting delivery of the carriers into organelles after internalization, especially endoplasmic reticulum (ER). For example, the researchers used small molecular peptide as a ligand of epidermal growth factor receptor to modify liposomes for tumor‐targeting. Lysosomal retention is the main limiting step for efficient gene transfection mediated by cationic liposomes. Several studies using peptide‐based cationic liposomes could obtain an enhanced transfection efficiency through lysosome escape. However, the main innovation of our material is that it can effectively avoid the capture of the carriers by lysosomes. Furthermore, pardaxin‐modified liposomes can deliver plasmid cargo to ER site close to the nucleus. The unique intracellular pathway of pardaxin‐modified liposomes could protect the plasmid cargo from degradation, prolong its retention in the cells, and make the plasmid closer to the nucleus, which may be the main reasons of highly efficient gene transfection mediated by the liposomes.

Herein, we report the development of a PAR‐modified cationic liposome to encapsulate the CRISPR/Cas9 system for efficient enhancement of gene editing efficiency. We have demonstrated that the PAR modification enables the cationic liposomes to easily escape capture by lysosomes and protects the DNA cargo from damage to enable further delivery of the cargo to the ER, inducing increased DNA entry into the nucleus. The accumulation of PAR‐Lipos in the ER may improve the binding of Cas9 and sgRNA, thus also contributing to enhanced gene editing. Given their high biosafety, PAR‐Lipos were used to mediate knockout of the oncogene CDC6 in vivo, which resulted in significant tumor growth inhibition. Our work may provide a useful reference for enhancing the delivery of gene editing systems, thus improving the potential for future clinical applications.

## Experimental Section

##### Reagents

DOTAP was purchased from Avanti Co. Ltd. (USA). 1,2‐Dioleoyl‐sn‐glycero‐3‐phosphoethanolamine (DOPE) and distearoyl‐sn‐glycero‐3‐phosphoethanolamine‐N‐[maleimide (polyethylene glycol)‐2000] (DSPE‐PEG_2000_) were obtained from AVT Co. Ltd. (Shanghai, China). A pardaxin peptide (HGFFALIPKIISSPLFKTLLSAVGSALSSSGGQE) was synthesized by Qiang Yao Biotech Co. Ltd. (Shanghai, China). 1‐Ethyl‐3‐(3‐dimethylaminopropyl) carbodiimide (EDC) and *N*‐hydroxy succinimide (NHS) were acquired from Sigma‐Aldrich Co. Ltd. (St Louis, MO). Di‐tert‐butyl dicarbonate ((BOC)_2_O) was obtained from Aladdin Co. Ltd. (Shanghai, China). Lipofectamine 2000 transfection reagent (Lipo2000) was purchased from Invitrogen Corp. (Carlsbad, CA). Dimethyl sulfoxide, methanol, and trichloroethane (CHCl_3_) were obtained from Sinopharm Chemical Reagent Co. Ltd. FITC and DiD perchlorate were provided by Meilun Biotech Co. Ltd. (Dalian, China). FITC‐labeled DNA was synthesized by Jierui Biotech Co. Ltd. (Shanghai, China). Hoechst 33 342, LysoTracker Red, ER‐Tracker Red, 3‐(4,5‐dimethylthiazol‐2‐yl)‐2,5‐diphenyltetrazolium bromide (MTT reagent), a Cell Cycle and Apoptosis Analysis Kit, and diethyl pyrocarbonate‐treated water (DEPC H_2_O, Dnase, and RNase free) were acquired from Beyotime Institute of Biotech (Jiangsu, China). A CDC6 antibody was procured from the Cell Signaling Technology Co. Ltd. (USA). An Animal Genomic DNA Kit and 2× High‐Fidelity Master Mix were obtained from Qingke Biotech Co. Ltd. (Beijing, China). A Quick Midi Purification Kit was purchased from Tiangen Biotech Co. Ltd. (Beijing, China). The deionized water used in all experiments was prepared using a Milli‐Q system (Millipore, Boston). All the chemicals and solvents were of analytical grade.

##### The Plasmid

A CRISPR/Cas9 editing plasmid system encoding mCherry (as a reporter gene), Cas9, and sgGFP (sgRNA targeting GFP, 5′‐CGGGTAGGACCAGCTCGACC‐3′), named pSpCas9‐sgGFP, was constructed with pX458. A CRISPR/Cas9 editing plasmid system encoding GFP (as a reporter gene), Cas9, and sgCDC6 (sgRNA targeting CDC6, 5′‐AGAGGCAGGGCTTTTACACG‐3′) was constructed and named pSpCas9‐sgCDC6.^[^ ^19^ ^]^ An EGFP plasmid (pEGFP) was obtained that only encoded the reporter gene EGFP. All plasmids were supplied by Sunny Biotech Co. Ltd.

##### Cell Culture and Animal Model

HEK293T cells (human embryonic kidney transformed cells), HEK293T‐GFP cells (HEK293T cells encoding the GFP gene), MCF‐7 cells (human breast carcinoma cells), and Huh7‐Luc cells (human liver carcinoma cells encoding the luciferase gene) were purchased from the Shanghai Cell Bank, Chinese Academy of Sciences. All cells were kept at 37 °C in a humidified atmosphere containing 5% CO_2_ and cultivated in Dulbecco's modified Eagle's medium (DMEM) containing 10% fetal bovine serum, 1% penicillin, and 1% streptomycin sulfate. All animal experiments were performed in accordance with the regulations of the Institutional Animal Care and Use Committee (IACUC) of Zhejiang University. Nude mice (5 weeks old, 18 ± 2 g) were raised under aseptic conditions in animal isolators with free access to food and water under a 12 h light/dark cycle. The animals were observed daily during the experiment.

##### Synthesis and Subcellular Localization of DSPE‐PEG_2000_‐PAR

DSPE‐PEG_2000_‐PAR was obtained through a reaction between DSPE‐PEG_2000_‐NH_2_ and the PAR peptide. First, the amino groups on PAR were protected with (BOC)_2_O. PAR (40 mg) was dissolved in 3 mL of anhydrous DMF, mixed with (BOC)_2_O at a molar ratio of 1:5.2, and stirred for 12 h in the dark at 4 °C. Then, EDC and NHS were added to the reaction mixture (PAR:EDC:NHS = 1:5:10, molar ratio), which was stirred for another 2 h to activate the carboxyl groups on PAR. Then, DSPE‐PEG_2000_‐NH_2_ (PAR: NH_2_‐DSPE‐PEG_2000_ = 1:1, molar ratio) was added, and the mixture was stirred for 24 h. To remove the protecting groups on PAR, 1 mL of HCl (12 m) was added. Afterward, the pH value was adjusted to 7.4 with NaOH (3 m). The reaction product was dialyzed with deionized water for 48 h and lyophilized to obtain DSPE‐PEG_2000_‐PAR. FITC‐labeled DSPE‐PEG_2000_‐PAR was further synthesized. Briefly, 5 mg of DSPE‐PEG_2000_‐PAR was reacted with 0.15 mg of FITC in 1 mL of DEPC‐treated water for 2 h at room temperature. The final product was purified by dialysis with pure water. MCF‐7 cells were incubated with FITC‐labeled DSPE‐PEG_2000_‐PAR (50 µg mL^−1^) for 2 h. Then, the cells were washed with PBS and subjected to nuclear, lysosomal, and ER staining with Hoechst 33 342, LysoTracker, and ER‐Tracker, respectively. The location of DSPE‐PEG_2000_‐PAR in cells was observed using confocal microscopy (A1R, Nikon, Japan).

##### Preparation and Characterization of PAR‐Lipos and Non‐Lipos

PAR‐Lipos and non‐Lipos (without PAR modification) were prepared through thin‐film hydration with ultrasonic dispersion. Briefly, DOTAP, DOPE, DSPE‐PEG_2000_‐NH_2_, and DSPE‐PEG_2000_‐PAR were dissolved in CHCl_3_, and a thin lipid film was obtained in a flask by solvent evaporation. PAR‐Lipos were prepared by hydration with DEPC‐treated water followed by probe ultrasonication. For preparation of non‐Lipos, DSPE‐PEG_2000_‐PAR was replaced with an equivalent molar amount of DSPE‐PEG_2000_‐NH_2,_ and non‐Lipos were obtained according to the same protocol mentioned above. The particle sizes and zeta potentials of the liposomes were measured (Malvern, UK). Their morphologies and structures were observed using TEM (JEOL JEM‐1230, Japan).

##### Toxicity

The cytotoxicity of Lipo2000, PAR‐Lipos, and non‐Lipos was evaluated in MCF‐7 and Huh7 cells using an MTT assay according to the manufacturer's suggested procedures. The cells were exposed to the various liposomes for 24 and 48 h. The data were expressed as the percentage of surviving cells and were reported as the mean values of five measurements. The effect of PAR‐Lipo on the viability of normal cells was further examined, and LO2 and HEK293T cells were selected as model cells. These cells were cultured with liposomes at transfection concentrations for 24 or 48 h. Then the cell viability was detected by Live‐Dead Cell Staining assay. The cytotoxicity of Lipo2000, PAR‐Lipos, and non‐Lipos after transfection was also tested on MCF‐7 cells. The dead cells were stained using propidium iodide in order to evaluate the transfection toxicity.

##### Agarose Gel Electrophoresis

Complexes of liposomes and plasmids were obtained by incubating the liposomes with pSpCas9‐sgGFP (at different mass ratios) at 37 °C for 30 min. Agarose gel electrophoresis of the complexes was carried out in a horizontal electrophoresis apparatus. The results were photographed using a gel imaging system (Bio‐Rad GelDoc‐EQ, USA).

##### EGFP Transfection In Vitro

The nucleic acid delivery capacities of Lipo2000, PAR‐Lipos, and non‐Lipos were investigated by GFP transfection. Briefly, complexes of liposomes and pEGFP (Lipo2000:pEGFP = 1.5:1, PAR‐Lipos:pEGFP = 5:1, non‐Lipos:pEGFP = 5:1, weight ratio, the final concentration [µg mL^−1^] of plasmid in the cell culture medium was 2) were prepared before transfection. MCF‐7 cells were incubated with the complexes in serum‐free DMEM for 4 h and then further incubated in complete medium for 24 or 48 h. EGFP fluorescence was observed using fluorescence microscopy, and the EGFP expression was presented as the relative level compared to that after 24 h of transfection using Lipo2000.

##### GFP Knockout In Vitro

Complexes of liposomes and pSpCas9‐sgGFP were obtained by incubating the liposomes with the plasmids (at different weight ratios) at 37 °C for 30 min. HEK293T‐GFP cells were incubated with the complexes in serum‐free DMEM for 4 h and then further incubated in complete medium for 24, 48, or 72 h. The GFP fluorescence intensity was quantified with a Cytomics FC 500 MCL (Beckman, USA). HEK293T‐GFP and HEK293T cells without any treatment were used as positive and negative controls, respectively. The percentage decrease of the GFP signal in each group compared with that in the positive control group was reported as the KO%. DNA was extracted using an Animal Genomic DNA Kit after 72 h of transfection, and then PCR amplification and purification were performed (forward: 5′‐TCAGCCTGCTTCTCGCTTCTG‐3′, reverse: 5′‐CTTGAAGAAGTCGTGCT GCTTCATG‐3′). Then, 200 ng of the resulting DNA was reannealed and digested with T7 endonuclease I (New England Biolabs) according to the manufacturer's protocol. Then, the samples were analyzed via 2% agarose gel electrophoresis. The DNA fragments were quantified on the basis of the relative band intensity using ImageJ software.

##### Cell Internalization and Subcellular Localization

DiD‐labeled liposomes were synthesized according to the method mentioned above. MCF‐7 cells were incubated with DiD‐labeled PAR‐Lipos or non‐Lipos (10 µg mL^−1^) for 0.5, 1, 2, or 4 h. The cell nuclei were stained with Hoechst 33 342, and the cells were fixed with 4% paraformaldehyde. The cellular internalization of the liposomes was observed using a fluorescence microscope (A1R, Nikon, Japan). Complexes were prepared by incubating PAR‐Lipos or non‐Lipos (14 µg) with a mixture of Cy3‐labeled DNA (0.5 µg) and a representative plasmid (pSpCas9‐sgGFP) (1.5 µg) at 37 °C for 30 min. MCF‐7 cells were incubated with the complexes for different times. The cellular uptake of the plasmid was observed using a fluorescence microscope and was analyzed using ImageJ software. To investigate the subcellular localization of the liposomes, cells were incubated with DiD‐labeled PAR‐Lipos or non‐Lipos (10 µg mL^−1^) for 1 h, and then the ER and lysosomes were stained with ER‐Tracker and LysoTracker, respectively. The colocalization of liposomes and organelles was determined using fluorescence microscopy. To investigate the effect of PAR‐Lipos on the intracellular behavior of sgRNA produced via the mediation of the CRISPR/Cas9 system, MCF‐7 cells were incubated with Cy3‐labeled DNA (to simulate sgRNA). Then, the cells were further treated with PAR‐Lipos or non‐Lipos (10 µg mL^−1^) for 0.5 and 1 h. The cells were washed with ice‐cold PBS, and the lysosomes and ER were stained with LysoTracker and ER‐Tracker, respectively. Cy3 fluorescence was observed using a fluorescence microscope.

##### Expression Analysis of CDC6

The expression levels of CDC6 in human breast carcinoma (BRCA) and human liver carcinoma (LIHC) were obtained from GEPIA (http://gepia.cancer‐pku.cn/). Survival curves of patients with different expression levels of the CDC6 gene were obtained from the TIMER (https://cistrome.shinyapps.io/timer/). The expression of CDC6 was further verified by western blot assay. Briefly, MCF‐7 and Huh‐7 cells were collected with CelLytic M cell lysis buffer containing protease inhibitor cocktail. The cell lysate was centrifuged at 12 000 rpm for 5 min, and the protein content in the supernatant was measured using an enhanced BCA Protein Assay Kit. The expression of CDC6 was determined by western blot analysis.

##### CDC6 Knockout In Vitro

Various complexes were prepared by incubating PAR‐Lipos, non‐Lipos, and Lipo2000 with pSpCas9‐sgCDC6 at 37 °C for 30 min (PAR‐Lipos:plasmid = 5–11:1). MCF‐7 cells were transfected with the complexes for 12 or 24 h. These cells were collected by centrifugation at 1000 rpm for 5 min. The cell sediment was treated with a Cell Cycle and Apoptosis Analysis Kit and analyzed with a Cytomics FC 500 MCL.

##### Biodistribution

Nude mice bearing MCF‐7 tumors were intravenously injected with DiD‐labeled PAR‐Lipos (20 mg kg^−1^). At the time points of 2, 4, 6, 12, 24, 48, and 72 h, the mice were observed with an in vivo imaging system (CRI, Co. Ltd., Woburn, MA) (Ex: 649 nm). The mice were sacrificed at 72 h post injection, and their major organs were collected and imaged.

##### Antitumor Effect In Vivo

Nude mice bearing MCF‐7 tumors (approximately 200 mm^3^) were randomly divided into four groups (six mice per group). The mice in groups 1–4 were intravenously injected once with saline. Naked pSpCas9‐sgCDC6 plasmids (1.25 mg kg^−1^), Lipo2000/pSpCas9‐sgCDC6 complexes (Lipo2000:plasmid = 1.5:1, weight ratio, 1.25 mg of pSpCas9‐sgCDC6 per kg), or PAR‐Lipo/pSpCas9‐sgCDC6 complexes (PAR‐Lipos:plasmid = 7:1, weight ratio, 1.25 mg of pSpCas9‐sgCDC6 per kg) were injected into the mice every three days. All tumor sizes (calculated as length × width × height/2) as well as body weight were monitored every other day. All mice were sacrificed on day 28 after the first injection, and the tumors were isolated, weighed, and further cut for H&E and Ki67 staining. Tumor tissues were also lysed, and the expression of CDC6 protein was determined by western blot analysis. Furthermore, an orthotopic liver cancer model was employed to test the antitumor effect of the CRISPR/Cas9 system mediated by PAR‐Lipos. Huh‐7‐Luc tumor blocks (2–3 mm^3^) were injected directly into the livers of nude mice through minimally invasive surgery. The liver cancer model was generated 14 days later. The mice were randomly divided into four groups (six mice per group) and received the same treatments as mentioned above. The body weight was monitored every 48 h. Luciferase bioluminescence was observed before and after various treatments using an IVIS Spectrum imaging system (Caliper, PerkinElmer). The mice were sacrificed on day 21 after the first treatment. Their organs were isolated for H&E staining. Bioluminescence intensity was reported as the average for each group.

##### Statistical Analysis

All the data displayed were representative of the results from multiple independent experiments. Data comparisons were performed with Student's *t‐*test and one‐way ANOVA. *p < 0.05 was regarded to indicate statistical significance. **p < 0.01 was considered to indicate extreme statistical significance. All error bars represented the ± SD.

## Conflict of Interest

The authors declare no conflict of interest.

## Supporting information

Supporting Information.
