# Supplementary material for: Appropriate Delivery of the CRISPR/Cas9 System through the Nonlysosomal Route: Application for Therapeutic Gene Editing
Source: Adv Sci (Weinh). 2020 Jun 13;7(14):1903381. doi: 10.1002/advs.201903381 (PMC7375254; doi:10.1002/advs.201903381)
Supplement: Supplementary file 1 — Supporting Information. [file ADVS-7-1903381-s001.pdf]

WILEY-VCH

Copyright WILEY-VCH Verlag GmbH & Co. KGaA, 69469 Weinheim, Germany, 2018.

Supporting Information

Appropriate Delivery of the CRISPR/Cas9 System through the Nonlysosomal Route: Application for Therapeutic Gene Editing

*Hang Yin, Xiaoling Yuan, Lihua Luo, Yichao Lu, Bing Qin, Junlei Zhang, Yingying Shi, Chunqi Zhu, Jie Yang, Xiang Li, Mengshi Jiang, Zhenyu Luo, Xinyu Shan, Dawei Chen, and Jian You**

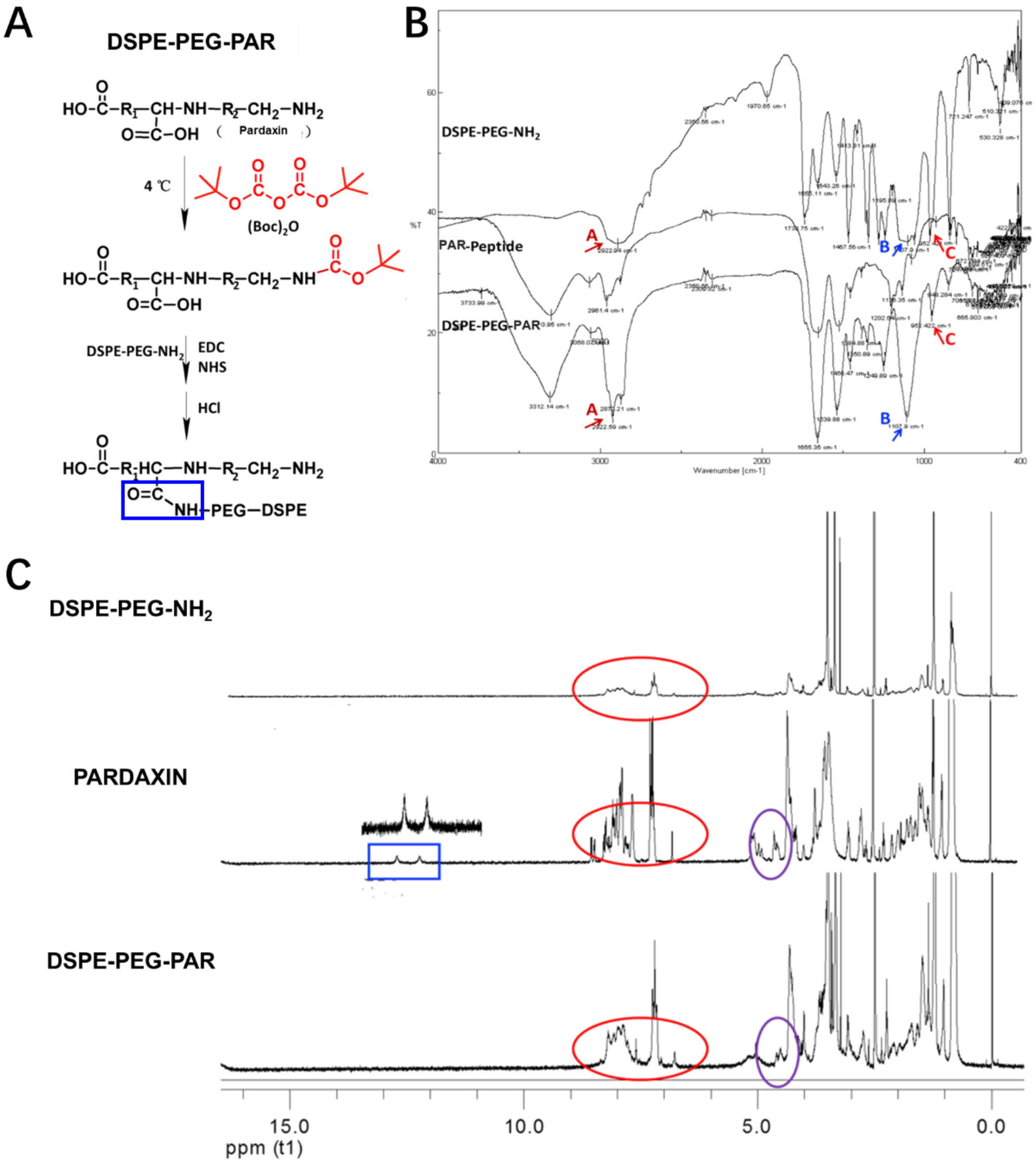

Figure S1. Synthesis and characterization of DSPE-PEG-PAR. (A) Organic synthetic route of DSPE-PEG-PAR. (B) The FT-IR spectrum of $DSPE\text{-}PEG\text{-}NH_2$, PARDAXIN, and DSPE-PEG-PAR, (A, ν C-H=2922.59 cm^{-1} B, ν C-O=1107.9 cm^{-1}, C, Δ C-H ($-CH_2$-

)=952.422 cm^{-1}) . (C) The ^{1}H-NMR (400 MHz, DMSO-d_6) of DSPE-PEG-NH_2, PARDAXIN, and DSPE-PEG-PAR. The signal in (C) corresponds to the structure in (A).

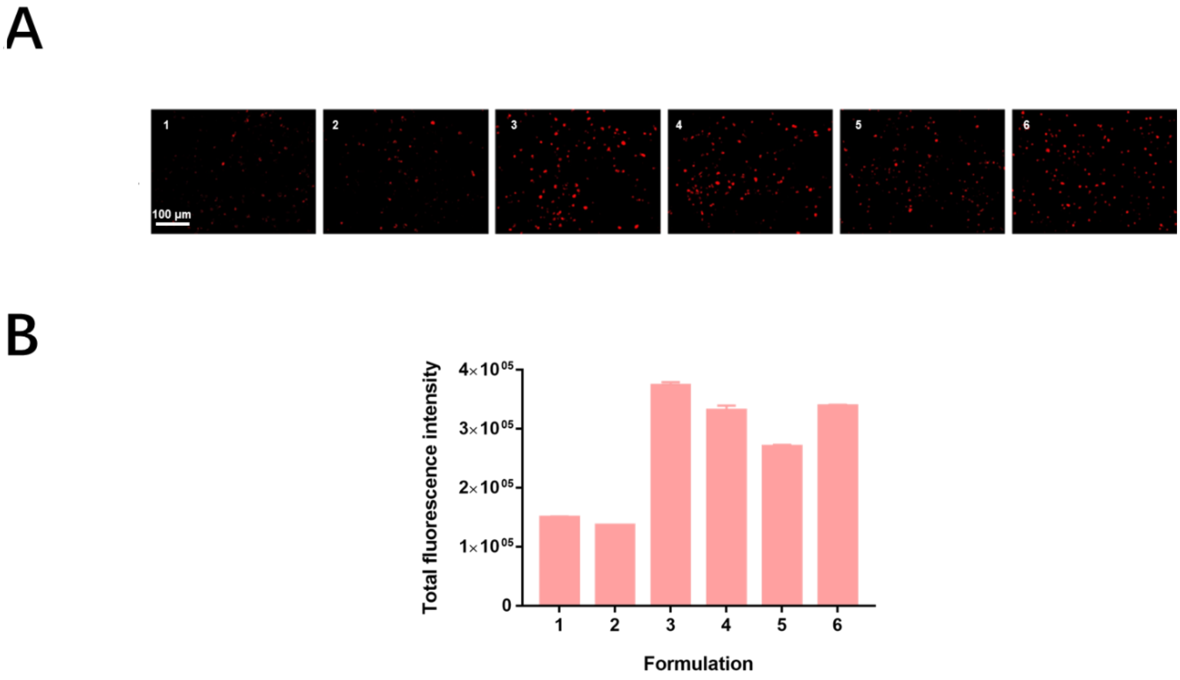

Figure S2. Screening of optimal prescriptions for transfection. (A) The cellular uptake efficiency of different Pardaxin-modified cationic liposome/DNA (Cy3 labeled) complexes (weight ratio: 5:1) and the total fluorescence intensity (B).

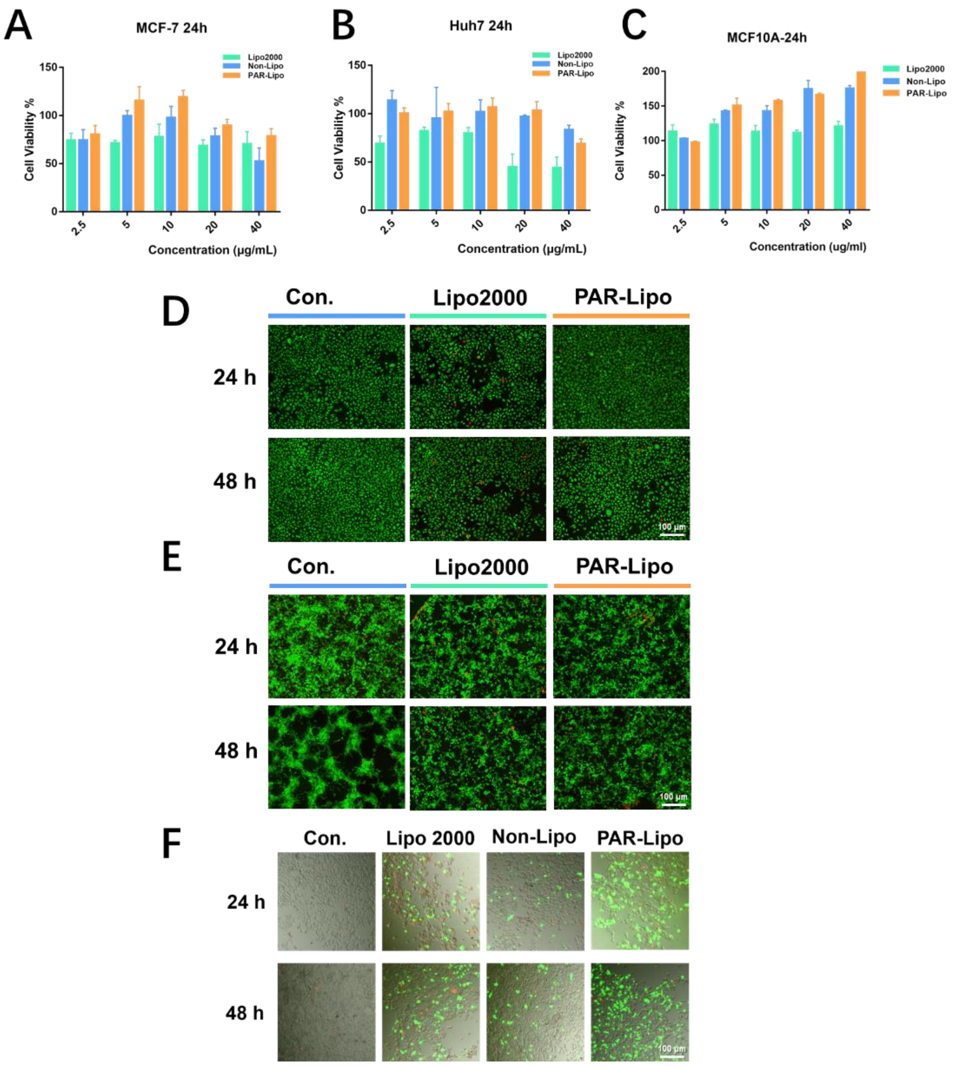

Figure S3. Cytotoxicity. Cytotoxicity of cationic liposomes to MCF-7 (A) , Huh7 (B) and MCF10A (C). The cytotoxicity of normal cells, LO2 (D) and HEK293T (E) was investigated using a live/dead staining method. Transfection cytotoxicity of cationic liposomes was evaluated by Propidium Iodide (PI) staining assay (F).

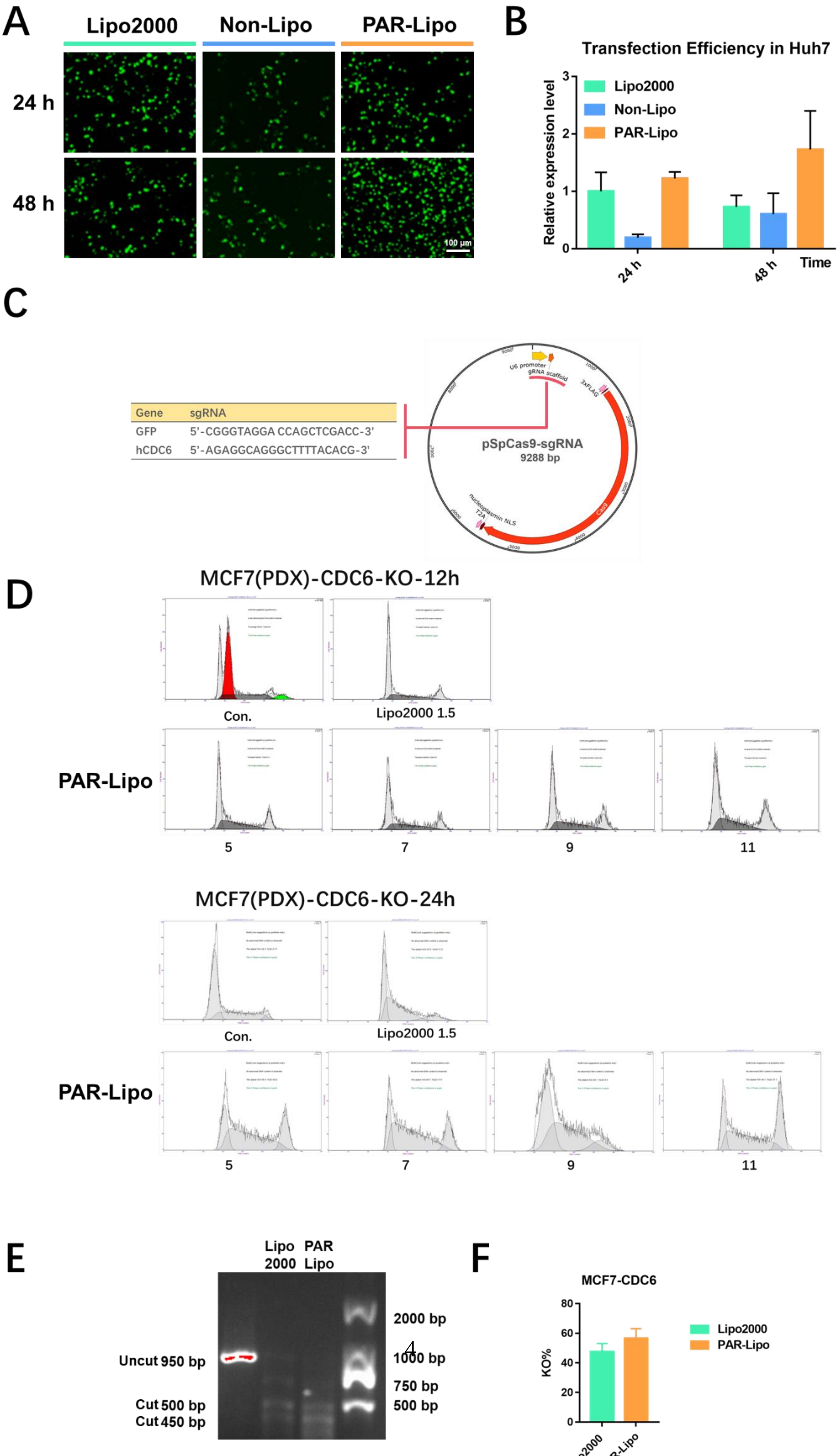
A
Lipo2000
Non-Lipo
PAR-Lipo
24 h
48 h
100 μm
B
Transfection Efficiency in Huh7
Relative expression level
Lipo2000
Non-Lipo
PAR-Lipo
24 h
48 h
Time
C
Gene sgRNA
GFP 5'-CGGGTAGGA CCAGCTCGACC-3'
hCDC6 5'-AGAGGCAGGGCTTTTACACG-3'
pSpCas9-sgRNA
9288 bp
U6 promoter
gRNA scaffold
3xFLAG
Cas9
nucleoplasmin NLS
T2A
D
MCF7(PDX)-CDC6-KO-12h
Con.
Lipo2000 1.5
PAR-Lipo
5
7
9
11
MCF7(PDX)-CDC6-KO-24h
Con.
Lipo2000 1.5
PAR-Lipo
5
7
9
11
E
Lipo 2000
PAR Lipo
Uncut 950 bp
Cut 500 bp
Cut 450 bp
2000 bp
1000 bp
750 bp
500 bp
F
MCF7-CDC6
KO%
Lipo2000
PAR-Lipo

Figure S4. The capacity of transfection in Huh7. (A) Green fluorescence proteins expression 24 h and 48 h after transfection by three cationic liposomes/pEGFP complexes to Huh7 cells. (B) The transfection efficiency semiquantitative analysis by ImageJ. (C) The plasmid was used to delivery CRISPR/Cas9 gene editing system. (D) The flow cytometry analysis images of MCF7-CDC6-KO. (E) Agarose gel electrophoresis results via T7E1 assay after 72 h transfection by Lipo2000/plasmid complexes (1.5:1) and PAR-Lipo/plasmid complexes (5:1). (F) Quantitative assay of agarose gel electrophoresis results using Imaging J software.

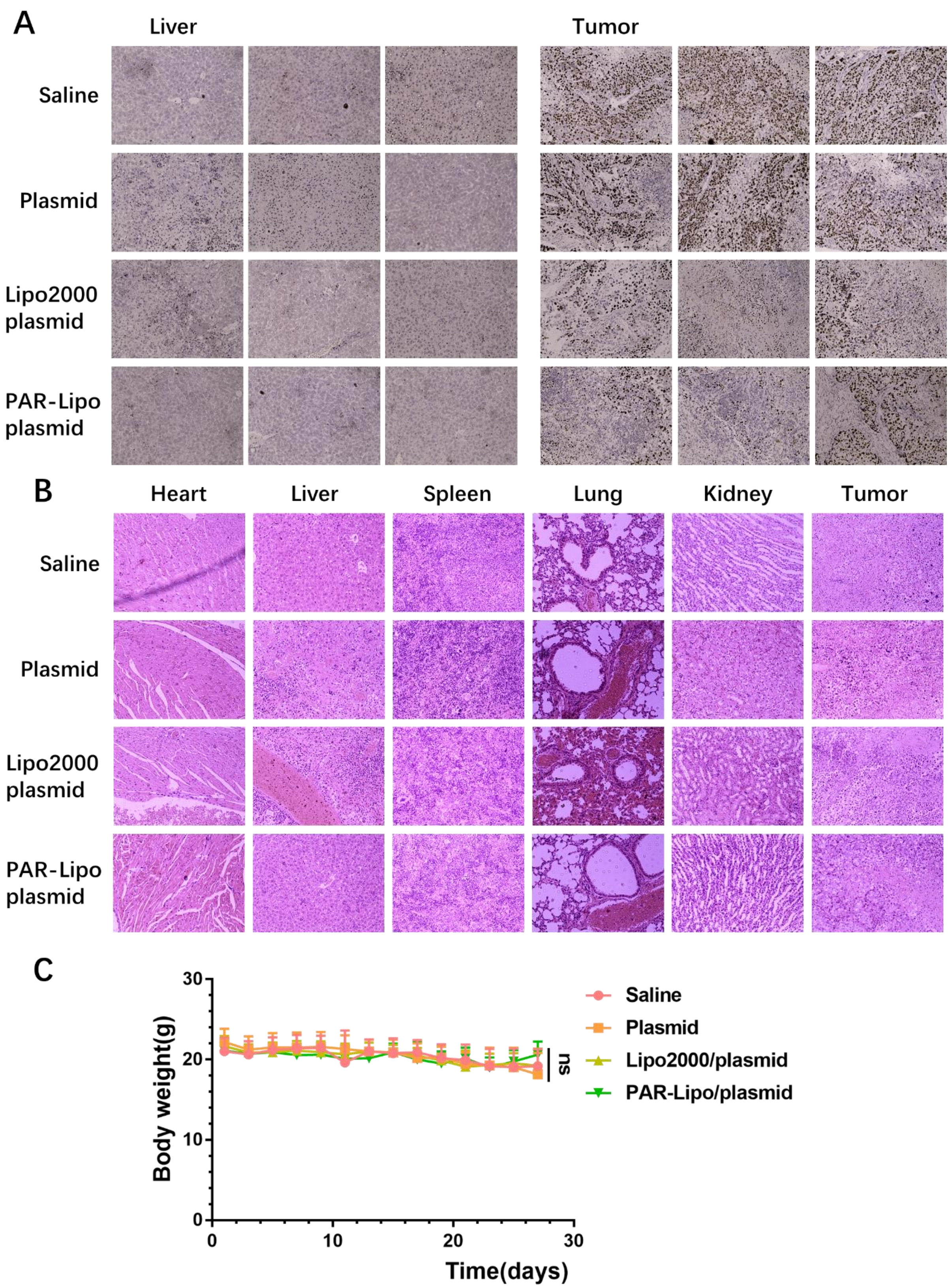

Figure S5. The safety of liposomes in MCF-7 tumor. Results of Ki67 (A) slices of tumor and liver in four groups of mice And H&E (B) slices of heart, liver, spleen, lung, kidney, tumor. (C) The changes of body weight during the treatment (ns means no statistical significance).

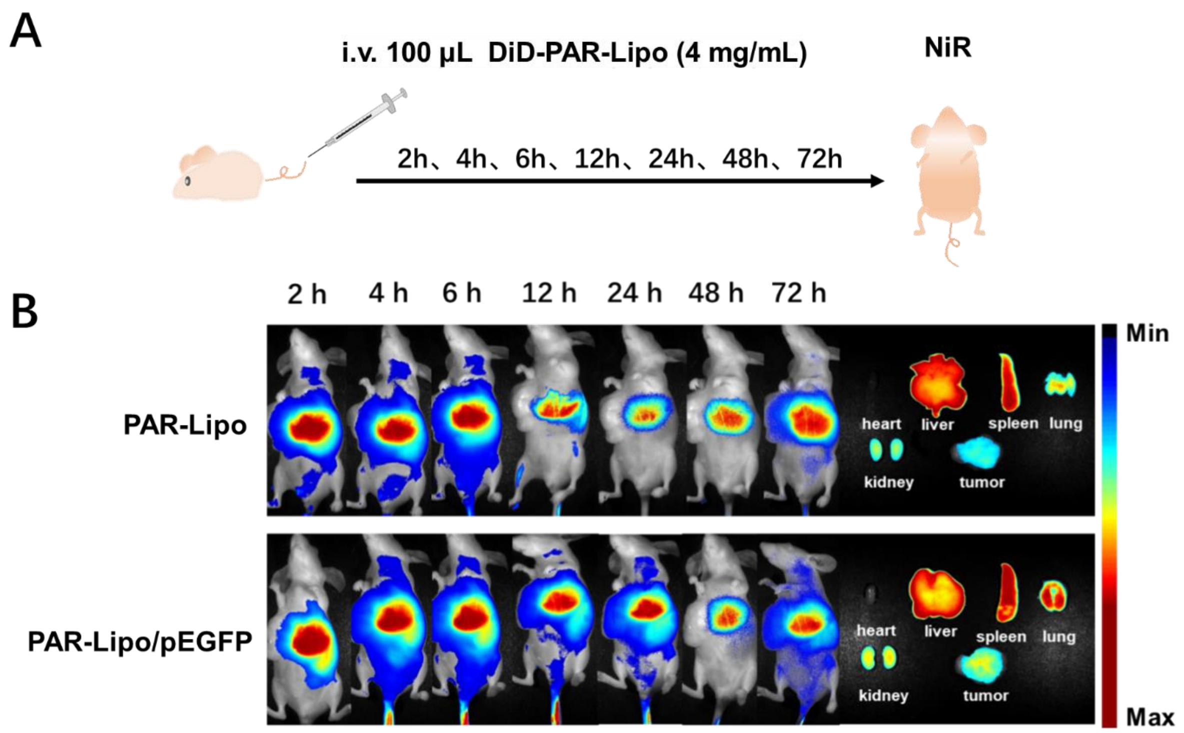

Figure S6. The distribution of PAR-Lipo and PAR-Lipo/DNA complexes *in vivo*. (A) Illustration of the liposomes administration scheme. (B) The bioluminescence images were obtained by IVIS spectrum imaging system.

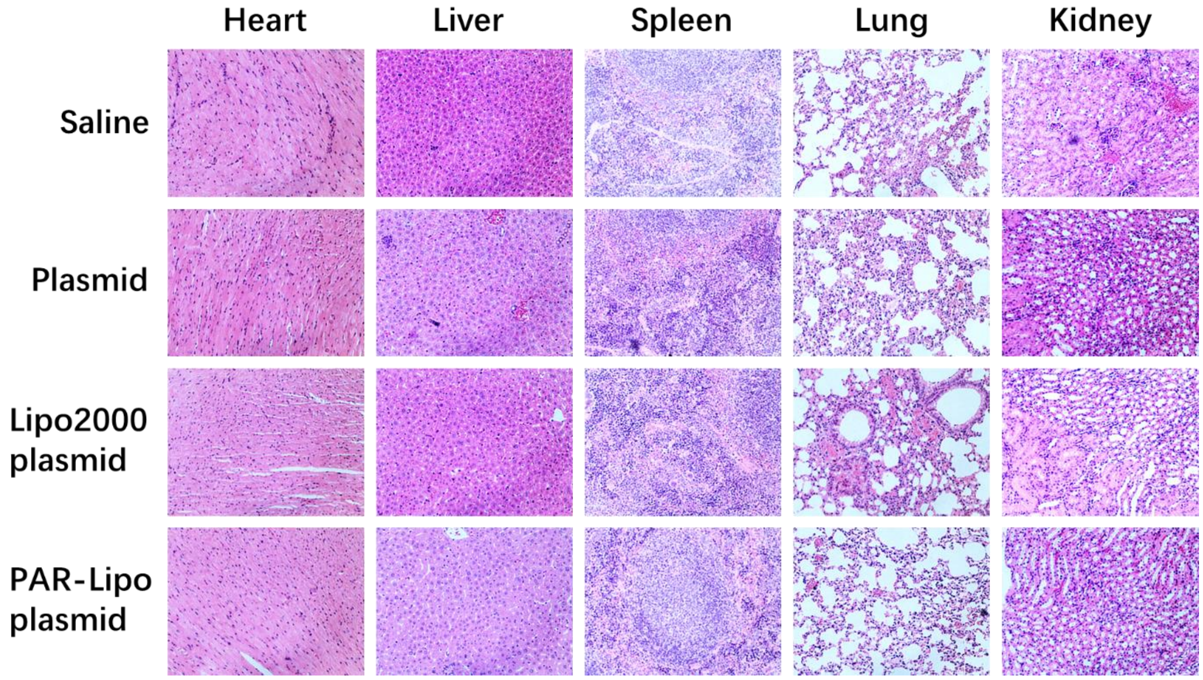

Figure S7. The safety of liposomes in Luci-Huh7 tumor. Results of H&E slices of heart, liver, spleen, lung, kidney, tumor.

Table S1. Six formulations of cationic liposomes modified with PAR peptide

Lipids (wt/wt)	Formulation					
	1	2	3	4	5	6

DOTAP : DOPE	2:1	2:1	4:1	4:1	8:1	8:1
DSPE-PEG-NH2 : DSPE-PEG-PAR	1:3	2:2	1:3	2:2	1:3	2:2

Table S2. Particle size and PDI (Polymer Dispersity Index) of PAR peptide modified liposomes and their DNA combined complexes

liposome : plasmid (wt/wt)		Formulation					
		1	2	3	4	5	6
liposome only	size (nm)	186.67	122	126.33	116	124.33	119.33
	PDI	0.219	0.35	0.373	0.400	0.368	0.386
5 : 1	size (nm)	219.33	135.33	140	133.67	165.33	245.67
	PDI	0.29	0.44	0.424	0.42	0.329	0.240
